# Resveratrol Modulates Phosphatidylcholine Metabolism-Related Enzymes and Enhances Chemosensitivity in Colorectal Cancer Models

**DOI:** 10.3390/ijms27156691

**Published:** 2026-07-27

**Authors:** Aurélie Mialhe, Elodie Mammar, Aline Mathey, Virginie Aires, Dominique Delmas

**Affiliations:** 1 Université Bourgogne Europe, INSERM, CTM UMR 1231, TIRECS Team, Bioactive Molecules and Health Research Group, 21000 Dijon, France; aurelie.mialhe1520@gmail.com (A.M.); elomammar@gmail.com (E.M.); aline.mathey52@gmail.com (A.M.); virginie.aires02@u-bourgogne.fr (V.A.); 2 Université Bourgogne Europe, Centre de Lutte Contre le Cancer G.-F. Leclerc, INSERM, CTM UMR 1231, TIRECS Team, Bioactive Molecules and Health Research Group, 21000 Dijon, France; 3 Université Bourgogne Europe, Centre de Lutte Contre le Cancer G.-F. Leclerc, Institut Agro, CHU Dijon Bourgogne, INSERM, BioSanD US 58, 21000 Dijon, France

**Keywords:** resveratrol, polyphenol, colorectal cancer, chemoresistance, lipid metabolism

## Abstract

Colorectal cancer (CRC) remains a major cause of cancer-related mortality worldwide, largely due to the emergence of chemoresistance driven by metabolic adaptations. Among these alterations, lipid metabolic reprogramming has emerged as a critical determinant of tumor progression and therapeutic response. In particular, dysregulation of enzymes involved in phosphatidylcholine (PC) synthesis and remodeling, through the Kennedy pathway and the Lands cycle, has been associated with therapeutic resistance. Lysophosphatidylcholine acyltransferase 2 (LPCAT2), a key enzyme involved in PC remodeling, notably promotes lipid droplets (LD) accumulation in CRC cells and contributes to chemoresistance. In this context, natural compounds capable of modulating PC metabolism, such as the polyphenol resveratrol (RSV), may represent a relevant strategy to improve sensitivity to chemotherapies in chemoresistant colon tumor cells. In this study, RSV induced apoptosis and significantly reduced the proliferation of intrinsically resistant HT29 cells and SW620 cells engineered to overexpress LPCAT2. RSV also decreased the expression of key enzymes involved in the Kennedy pathway in chemoresistant CRC cell lines. These changes were accompanied by distinct time-dependent patterns of lipid droplet accumulation in the two cellular models. In addition, drug combination studies showed that RSV enhanced the response to 5-fluorouracil (5-FU), oxaliplatin (OXA) and the FOX regimen (5-FU + OXA, 1:1 ratio) with synergistic interactions for several dose combinations, together with favorable dose-reduction indices. Collectively, these in vitro findings indicate that RSV modulates PC-metabolism-related proteins and enhances chemotherapy sensitivity in resistant CRC models. Overall, our results identify RSV as a promising therapeutic strategy to target metabolic vulnerabilities associated with PC metabolism and overcome chemoresistance in CRC.

## 1. Introduction

Cancer cells undergo extensive metabolic rewiring to sustain uncontrolled proliferation, adapt to cellular stress, and evade therapeutic pressure. Among these alterations, lipid metabolic reprogramming has emerged as a hallmark of cancer, influencing membrane biogenesis, energy storage, and oncogenic signaling pathways [1,2]. This process is largely orchestrated by Sterol Regulatory Element-Binding Proteins (SREBPs), which promote de novo lipogenesis through the activation of enzymes such as ATP citrate lyase (ACLY), acetyl-CoA carboxylase (ACC), and fatty acid synthase (FASN), while simultaneously enhancing lipid uptake and storage [3]. Beyond fatty acid synthesis, the remodeling of complex lipids, particularly phospholipids, plays a critical role in regulating membrane architecture and signaling nanodomains involved in oncogenic pathways, including RAS–RAF–MEK–extracellular signal-related kinase (ERK) and protein kinase C (PKC) [4]. These lipid-driven processes actively regulate cell fate decisions, stress adaptation, and therapeutic sensitivity, thereby positioning lipid metabolism as a critical regulator of cancer progression and treatment response [2,5]. Among membrane phospholipids, phosphatidylcholine (PC) is the most abundant species, accounting for approximately 40–50% of total cellular phospholipids. Beyond its structural role, PC serves as a precursor for several bioactive lipid mediators, including diacylglycerol (DAG), lysophosphatidylcholine (LPC), and phosphatidic acid (PA) [4]. These metabolites act as second messengers that potentiate mitogenic, inflammatory, and survival signaling pathways, thereby contributing to tumor progression and chemoresistance. PC homeostasis is maintained through two major metabolic pathways: the Kennedy pathway, responsible for de novo PC biosynthesis through the sequential activities of choline kinase α (CKα), CTP:phosphocholine cytidylyltransferase α (CCTα), and choline phosphotransferase 1 (CHPT1), and the Lands cycle, which mediates PC remodeling through the reacylation of LPC via lysophospholipid acyltransferases (LPCATs) [6]. Dysregulation of these pathways has been strongly associated with tumor aggressiveness and resistance to anticancer therapies. In colorectal cancer (CRC), alterations in choline metabolism correlate with prognosis, immune infiltration, and chemotherapy response, underscoring the clinical relevance of PC biosynthesis and remodeling [5,7].

Recent evidence has identified LPCAT2, a key enzyme of the Lands cycle, as a major determinant of chemoresistance in CRC. In this context, we previously demonstrated that LPCAT2 promotes lipid droplet (LD) biogenesis, thereby buffering lipotoxic stress and impairing apoptotic signaling, ultimately reducing immunogenic cell death and limiting CD8^+^ T-cell infiltration in tumors exposed to 5-fluorouracil (5-FU) and oxaliplatin (OXA) [8,9]. These findings support LPCAT2 as a potential metabolic vulnerability and suggest that modulating PC remodeling may help restore chemosensitivity. This concept is further supported by the emerging role of LD in cancer biology. Once considered inert lipid storage organelles, LD are now recognized as highly dynamic structures involved in lipid storage, membrane synthesis, and cellular adaptation. Their biogenesis depends on PC availability to ensure phospholipid monolayer stability, thereby directly linking PC metabolism to LD dynamics and cancer cell survival under stress conditions [10,11]. In CRC and several other malignancies, LD accumulation has been associated with aggressive phenotypes and poor therapeutic response, highlighting LD-associated pathways as attractive therapeutic targets [11]. 

In parallel, phospholipid alterations have emerged as promising biomarkers in CRC. Lipidomic analyses have consistently identified PC, phosphatidylethanolamine (PE), ceramides and triacylglycerols (TAG) among the lipid classes most significantly dysregulated in CRC tissues and patient plasma samples [12]. Moreover, extracellular vesicles derived from CRC patients display stage-dependent alterations in specific PC and PE species that mirror changes observed in CRC cellular models [13]. Beyond case–control studies, prospective analyses from the European Prospective Investigation into Cancer and Nutrition (EPIC) cohort reported inverse associations between selected hydroxysphingomyelin and ether-linked PC species and CRC risk, suggesting that pre-diagnostic lipid signatures may reflect carcinogenic trajectories [14]. Collectively, these findings support phospholipid metabolism as a clinically relevant framework for the identification of biomarkers and therapeutic targets while emphasizing the need for standardized analytical approaches and large-scale validation studies [15].

This metabolic context provides a strong rationale for exploring bioactive dietary compounds as modulators of cancer lipid metabolism. Polyphenols, a broad class of plant-derived bioactive molecules, have attracted considerable interest owing to their antioxidant, anti-inflammatory, and immunomodulatory properties, as well as their ability to interfere with oncogenic signaling pathways and sensitize tumors cells to therapy [16,17]. Among them, resveratrol (RSV), a naturally occurring polyphenol enriched in grapes, has been extensively investigated for its pleiotropic anticancer properties, including antioxidant, anti-inflammatory, pro-apoptotic, and chemosensitizing activities [18,19,20]. RSV modulates several signaling pathways involved in tumor progression, including nuclear factor-kappa B (NF-κB), signal transducer and activator of transcription 3 (STAT3), and mitogen-activated protein kinase (MAPK)/ERK signaling, and sensitizes tumor cells to death receptor-mediated apoptosis by promoting death-inducing signaling complex (DISC) formation [21,22]. Clinical studies have demonstrated its safety at doses up to 1 g/day and suggested beneficial metabolic effects, although robust evidence supporting its efficacy in oncology remains limited [23]. Importantly, RSV has also been shown to interfere with lipid metabolism, notably through the modulation of SREBP-dependent lipogenesis [24,25].

Despite these observations, whether RSV directly regulates PC metabolism in CRC by targeting both the Kennedy pathway and LPCAT2-mediated PC remodeling remains poorly understood. Therefore, we investigated the effects of RSV on the expression of key enzymes involved in the Kennedy pathway and LPCAT2-mediated PC remodeling in chemoresistant CRC models, including HT29 and SW620 cells overexpressing LPCAT2, and evaluated whether RSV could enhance their response to conventional chemotherapeutic agents. In the present study, we demonstrate that RSV reduces cell viability and induces apoptosis in intrinsically resistant HT29 cells and in SW620-lpcat2 cells. Notably, RSV disturbs the expression of key enzymes involved in the Kennedy pathway and modulates LPCAT2 expression in a cell-context-dependent manner. These molecular changes were accompanied by distinct time-dependent patterns of lipid droplet (LD) accumulation in the two cellular models. Furthermore, RSV pretreatment enhances the response of resistant HT29 cells and metastatic SW620 cells overexpressing LPCAT2 to several chemotherapeutic agents, including 5-fluorouracil (5-FU), oxaliplatin (OXA) and the FOX (5-FU + OXA, 1:1 ratio) regimen. Collectively, our findings provide mechanistic insights into the ability of RSV to modulate PC metabolism and potentiate therapeutic sensitivity in colorectal cancer.

## 2. Results

### 2.1. Resveratrol Impairs the Proliferation of Colorectal Cancer (CRC) Cells

**Figure 1:**
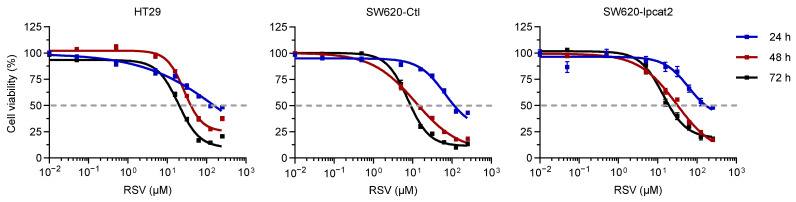
Resveratrol (RSV) reduces the viability of colorectal cancer cells. HT29, SW620-Ctl, and SW620-lpcat2 cells were treated with increasing concentrations of RSV (0–250 µM; ethanol solvent) for 24, 48, and 72 h. Cell viability was assessed by crystal violet staining. Results are expressed as percentage of viable cells relative to untreated controls and presented as mean ± SEM from three independent experiments (*n* = 6 per condition). The concentration inducing 50% cell death (IC_50_) was determined at each time point using four-parameter nonlinear regression analysis. The horizontal dashed line indicates 50% cell viability and represents the threshold used to determine IC_50_ values.

Colorectal cancer (CRC) cells display profound alterations in lipid metabolism [15,26]. In particular, increased LPCAT2 expression promotes phosphatidylcholine (PC) remodeling and lipid droplet (LD) biogenesis, two key features previously associated with chemoresistance [8]. Our previous studies also demonstrated marked differences in the sensitivity of CRC cell lines to both resveratrol (RSV) [22] and conventional chemotherapy [8]. Among these models, HT29 cells exhibit the highest endogenous LPCAT2 expression and LD accumulation and are among the most resistant, whereas SW620 cells display the lowest LPCAT2 expression and LD content and are among the most sensitive. Based on these well-established differences, HT29 cells were selected as a model of intrinsic resistance, while SW620 cells engineered to overexpress LPCAT2 (SW620-lpcat2) were used as an isogenic model of LPCAT2-driven acquired resistance. The corresponding SW620-Ctl cells served as the appropriate control. Using these complementary models, we investigated whether RSV retains its antiproliferative activity in both intrinsic and acquired resistance settings and whether this response is associated with LPCAT2-dependent PC metabolism. HT29, SW620-Ctl and SW620-lpcat2 cells were therefore exposed to increasing concentrations of RSV for 24, 48 and 72 h. Cell viability was determined by crystal violet staining. Dose–response curves were generated and half-maximal inhibitory concentrations (IC_50_) were calculated for each cell line at each time point (Figure 1 and Table 1).

RSV induced a marked, time and dose-dependent decrease in cell viability in all CRC models tested (Figure 1). HT29 cells, despite their intrinsic chemoresistant phenotype, displayed progressive sensitivity to RSV over time, with IC_50_ values decreasing from 157.4 ± 5.38 µM at 24 h to 22.39 ± 2.63 µM after 72 h of treatment (Table 1). Similarly, SW620-lpcat2 cells, which display enhanced LD accumulation and chemoresistance associated with LPCAT2 overexpression, became increasingly sensitive to RSV upon prolonged exposure, with IC_50_ values decreasing from 169.5 ± 20.31 µM at 24 h to 28.39 ± 12.10 µM at 72 h. SW620-Ctl cells were the most sensitive to RSV treatment at all time points, exhibiting IC_50_ values of 13.01 ± 1.57 µM and 8.75 ± 1.77 µM after 48 h and 72 h of treatment, respectively (Figure 1 and Table 1). These growth inhibitory effects were observed at moderate RSV concentrations, suggesting that the reduction in cell viability was not solely attributable to nonspecific cytotoxicity. Importantly, despite their intrinsic or LPCAT2-driven chemoresistant phenotypes, both resistant models remained responsive to prolonged RSV exposure, with a marked increase in sensitivity over time. Overall, these findings demonstrate that RSV effectively reduces cell viability in both intrinsic and LPCAT2-driven models of chemoresistant CRC in a concentration- and time-dependent manner.

### 2.2. Resveratrol Induces Apoptosis in Chemoresistant HT29 and LPCAT2-Overexpressing SW620 Colorectal Cancer Cells

Following the marked reduction in cell viability induced by RSV, we next investigated whether this effect was associated with apoptosis. Because the objective of this study was to evaluate RSV activity in two complementary models of chemoresistance, all subsequent analyses were performed independently within each model. HT29 and SW620-lpcat2 cells were therefore considered as representative models of intrinsic and LPCAT2-driven acquired resistance, respectively, rather being directly compared as equivalent cellular systems. Apoptosis was evaluated after 24, 48, and 72 h of RSV treatment, by Annexin V/7-AAD staining and immunoblotting of key proteins involved in late apoptotic events.

**Figure 2:**
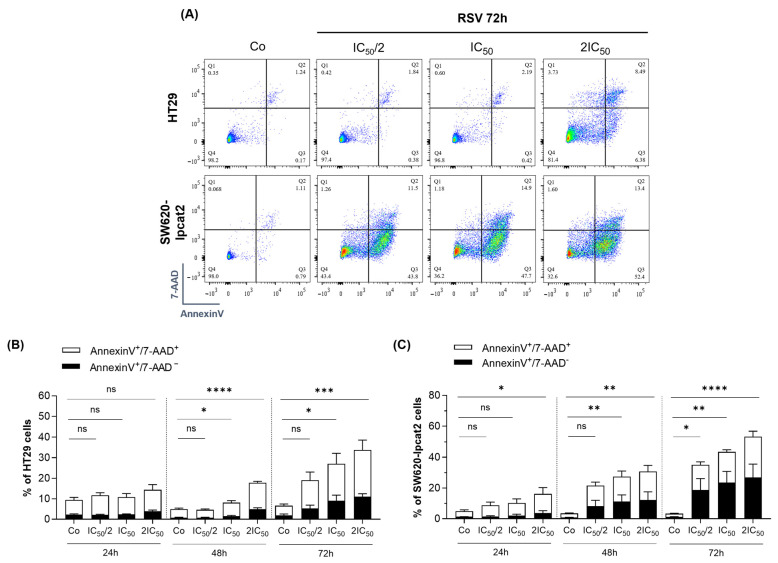
RSV induces apoptosis in HT29 and SW620-lpcat2 CRC cell lines. After 24 h of culture, SW620-lpcat2 and HT29 cells were treated with vehicle control (0.1% Ethanol; Co) or increasing concentrations of RSV (IC_50_/2, IC_50_, 2xIC_50_; IC_50 HT29_ = 22.39 µM; IC_50 SW620-lpcat2_ = 28.39 µM) for 24, 48 and 72 h. (**A**) Representative AnnexinV/7-AAD flow cytometry dot plots obtained after 72 h of treatment with the indicated RSV concentrations. Each dot represents an individual cellular event, and the color scale reflects event density, ranging from blue (low density) to red (high density). AnnexinV and 7-AAD fluorescence intensities are displayed on the horizontal and vertical axes, respectively. (**B**,**C**) Quantification of early apoptotic cells (AnnexinV-positive/7AAD-negative population, white bars) and late apoptotic cells (AnnexinV-positive/7AAD-positive population, black bars) following RSV treatment for 24 h, 48 h and 72 h in HT29 cells (**B**) and SW620-lpcat2 cells (**C**). Data are presented as mean ± SEM from three independent experiments. Statistical analyses were performed using either two-way ANOVA, followed by Tukey’s multiple comparison test. ns, not significant; * *p* < 0.05, ** *p* < 0.01, *** *p* < 0.001, **** *p* < 0.0001.

HT29 and SW620-lpcat2 cells were treated with increasing RSV concentrations corresponding to the IC_50_/2, IC_50_, and 2xIC_50_ (2IC_50_) for 24, 48 and 72 h prior to AnnexinV/7-AAD staining. Flow cytometry analyses revealed that RSV induced apoptosis in both resistance models in a concentration- and time-dependent manner (Figure 2). Representative AnnexinV/7-AAD flow cytometry dot plots obtained after 72 h of treatment are shown in Figure 2A. Each dot represents an individual cellular event, and the color scale indicates event density, ranging from blue (low density) to red (high density). AnnexinV and 7-AAD fluorescence intensities are displayed on the horizontal and vertical axes, respectively. Viable cells (AnnexinV^−^/7-AAD^−^) are located in the lower left quadrant, whereas early apoptotic (AnnexinV^+^/7-AAD^−^), late apoptotic (AnnexinV^+^/7-AAD^+^) and necrotic (AnnexinV^−^/7-AAD^+^) cells are distributed in the lower right, upper right and upper left quadrants, respectively. Quantitative analyses confirmed that, in the intrinsic resistance model (HT29), RSV induced a concentration- and time-dependent increase in apoptotic cell populations (Figure 2A,B). Apoptosis became more pronounced after 48 h and further increased after 72 h, particularly at IC_50_ and 2IC_50_. A similar pattern was observed in the LPCAT2-driven acquired resistance model (SW620-lpcat2) (Figure 2C). Both early and late apoptotic cell populations increased following RSV treatment, with apoptosis becoming apparent after 48 h and further increasing after 72 h. Notably, apoptotic cells were already detectable at IC_50_/2 after 72 h, with a further increase at IC_50_ and 2IC_50_.

**Figure 3:**
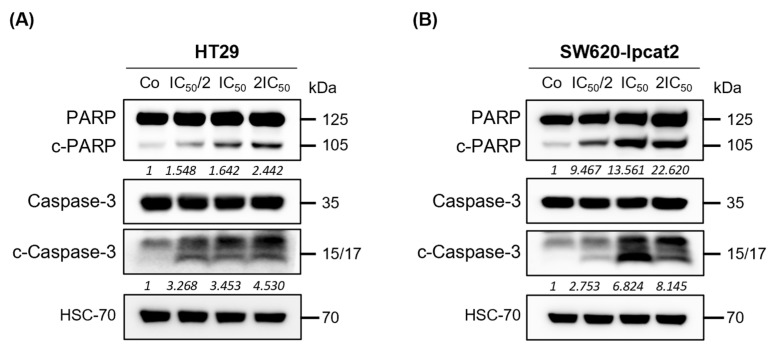
RSV cleaves caspase-3 and PARP proteins in HT29 and SW620-lpcat2 cell lines. Representative immunoblot of apoptosis-related proteins following 72 h of RSV treatment in HT29 (**A**) and SW620-lpcat2 cells (**B**). Pro- and cleaved forms of caspase-3 and PARP are shown. HSC-70 was used as loading control. Densitometric quantification of cleaved caspase-3 and cleaved PARP levels.

To further confirm apoptosis induction, activation of the execution phase of apoptosis was evaluated by immunoblotting through the analysis of caspase-3 and poly(ADP-ribose) polymerase (PARP) cleavage after 72 h of RSV treatment (Figure 3). In both resistance models, RSV induced dose-dependent cleavage of procaspase-3 and PARP, confirming activation of the apoptotic cascade.

### 2.3. Resveratrol Alters the Expression of Key Enzymes Involved in Phosphatidylcholine Metabolism in Colorectal Cancer Cells

**Figure 4:**
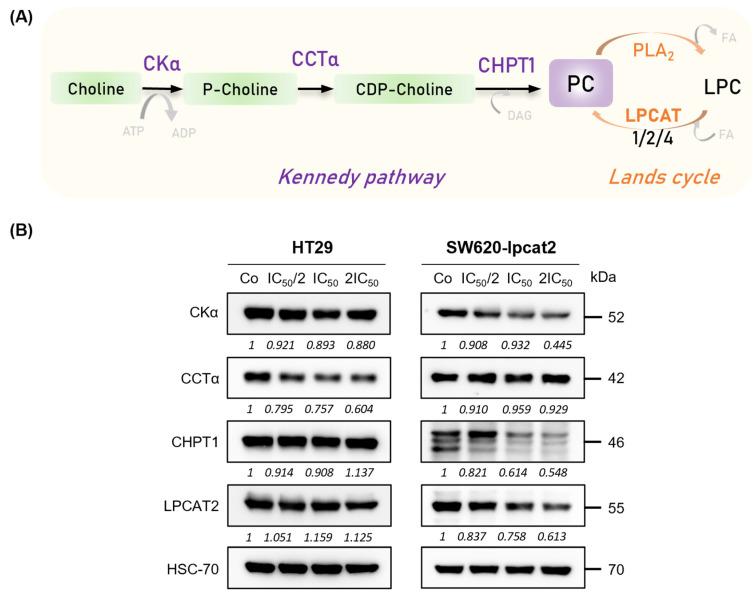
RSV decreases the expression of key enzymes involved in PC metabolism in CRC cells. (**A**) Schematic representation of the Kennedy pathway and Lands cycle involved in phosphatidylcholine (PC) biosynthesis and remodeling. (**B**) Representative immunoblot analysis of CKα, CCTα, CHPT1 and LPCAT2 in HT29 and SW620-lpcat2 cells treated with increasing concentrations of resveratrol (RSV; IC_50_/2, IC_50_, and 2IC_50_) for 72 h. HSC-70 was used as loading control. Abbreviations: CKα, choline kinase α; CCTα, CTP:phosphocholine cytidylyltransferase α; CHPT1, choline phosphotransferase 1; FA, fatty acid; LPCAT, lysophosphatidylcholine acyltransferase; PLA_2_, phospholipase A_2_; DAG, diacylglycerol; LPC, lysophosphatidylcholine; PC, phosphatidylcholine.

Given the marked effects of RSV on CRC cell viability and apoptosis, we next investigated whether these responses were associated with alterations in key regulators of PC metabolism. PC homeostasis involves two major metabolic pathways: the Kennedy pathway, which mediates de novo PC biosynthesis through the sequential activities of choline kinase α (CKα), CTP:phosphocholine cytidylyltransferase α (CCTα), and choline phosphotransferase 1 (CHPT1), and the Lands cycle, which regulates PC remodeling through lysophospholipid acyltransferases, including a critical enzyme LPCAT2 (Figure 4A) [6,27]. Notably, we recently identified CHPT1, the enzyme catalyzing the final step of the Kennedy pathway, as a central metabolic regulator of chemoresistance in CRC, highlighting the terminal step of the Kennedy pathway as a key determinant of drug response [26]. Because these pathways are frequently dysregulated in cancer and contribute to tumor progression and therapeutic resistance, we evaluated whether RSV could modulate the expression of enzymes involved in PC biosynthesis and remodeling.

To this end, HT29 and SW620-lpcat2 cells were treated with increasing concentrations of RSV for 72 h, and the expression of CKα, CCTα, CHPT1, and LPCAT2 was analyzed by immunoblotting (Figure 4B). RSV treatment induced marked alterations in the expression of enzymes across the different CRC models. In HT29 cells, RSV primarily affected enzymes of the Kennedy pathway. CCTα expression decreased in a concentration-dependent manner, reaching 0.757- and 0.604-fold of control levels at IC_50_ and 2IC_50_, respectively, whereas CKα expression showed a more moderate reduction. In contrast, CHPT1 and LPCAT2 expression remained largely unchanged following RSV treatment (Figure 4B). In SW620-lpcat2 cells, RSV strongly decreased both CKα and CHPT1 expression in a concentration-dependent manner, reaching 0.445-fold and 0.548-fold of control levels, respectively, following treatment with 2IC_50_ RSV (Figure 4B). Interestingly, RSV differentially modulated the expression of enzymes involved in PC metabolism according to the cellular context. In HT29 cells, RSV predominantly affected enzymes of the Kennedy pathway, whereas in SW620-lpcat2 cells, it also reduced LPCAT2 expression in a concentration-dependent manner (Figure 4B). These findings indicate that the pattern of RSV-induced modulation of PC metabolic enzymes differs between the two cellular models.

### 2.4. Resveratrol Promotes Lipid Droplet Accumulation in SW620-lpcat2 CRC Cells

**Figure 5:**
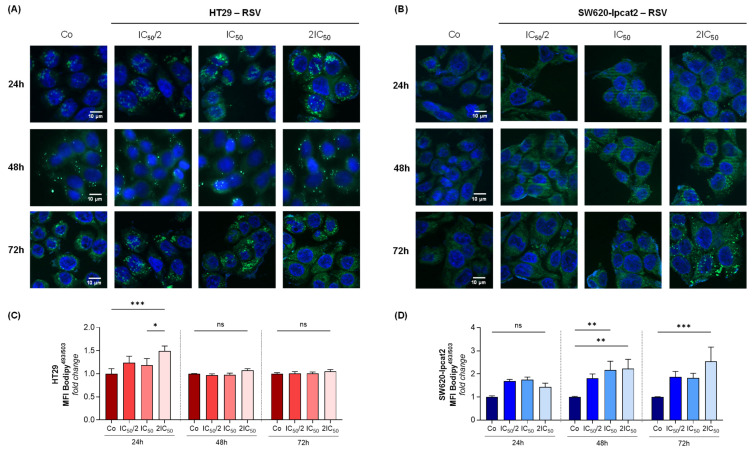
RSV modulates LD accumulation in HT29 and SW620-lpcat2 CRC cells. Cells were treated with vehicle control (Co) or increasing concentrations of resveratrol (RSV) corresponding to the 72 h-IC_50_ values (22.39 µM for HT29 and 28.39 µM for SW620-lpcat2) for 24, 48 and 72 h. (**A**,**B**) Representative fluorescence microscopy images of intracellular lipid droplet (LD) in HT29 cells (**A**) and SW620-lpcat2 cells (**B**). LD were stained with BODIPY 493/503 (green) and nuclei with DAPI (blue). Scale bar = 10 µm. (**C**,**D**) Quantification of LD accumulation in HT29 (**C**) and SW620-lpcat2 (**D**) cells, determined by measuring the median fluorescence intensity (MFI) of BODIPY 493/503 in live cells (FVS700-negative population). Results are expressed as fold change relative to the corresponding vehicle-treated control and are presented as mean ± SEM from three independent experiments. Statistical analyses were performed using two-way ANOVA followed by Tukey’s multiple comparison test. ns, not significant; * *p* < 0.05, ** *p* < 0.01, *** *p* < 0.001.

Given the established role of LPCAT2 in lipid droplet (LD) biogenesis [8], and its differential modulation by RSV (Figure 4B), we next investigated whether RSV also affected intracellular LD accumulation. LD are dynamic lipid storage organelles involved in cellular adaptation to metabolic stress and have been associated with chemoresistance in CRC cells [8]. To address this, HT29 and SW620-lpcat2 cells were treated with RSV for 24, 48 and 72 h and stained with BODIPY 493/503 to visualize intracellular LD by fluorescence microscopy (Figure 5A,B) and quantify LD content over time by flow cytometry (Figure 5C,D).

In HT29 cells, RSV induced a modest and transient increase in LD accumulation that was primarily observed after 24 h of treatment and progressively declined following prolonged exposure (Figure 5A,C). Consistent with these observations, no significant changes in LD content were detected after 48 or 72 h of treatment. In SW620-lpcat2 cells, RSV induced a progressive concentration-dependent increase in LD accumulation, which became more pronounced with treatment duration (Figure 5B,D). Fluorescence microscopy confirmed the increased number of intracellular LD following RSV treatment (Figure 5A,B). 

Overall, these findings indicate that RSV differentially modulates LD accumulation according to the cellular context, in parallel with the cell-specific changes observed in the expression of PC-metabolism-related proteins (Figure 4). These LD profiles may reflect different adaptive responses to RSV-induced metabolic stress. In SW620-lpcat2 cells, the sustained increase in LD content occurred concomitantly with reduced expression of LPCAT2 and selected Kennedy pathway enzymes. Although no causal relationship can be established from the present data, this response may reflect a redistribution of cellular lipids toward neutral lipid storage in response to altered phospholipid homeostasis. Further lipidomic and functional analyses will be required to determine whether these changes are directly associated with alterations in PC turnover and LD metabolism.

### 2.5. Resveratrol Sensitizes Chemoresistant CRC Cells to Conventional Chemotherapeutic Agents

**Figure 6:**
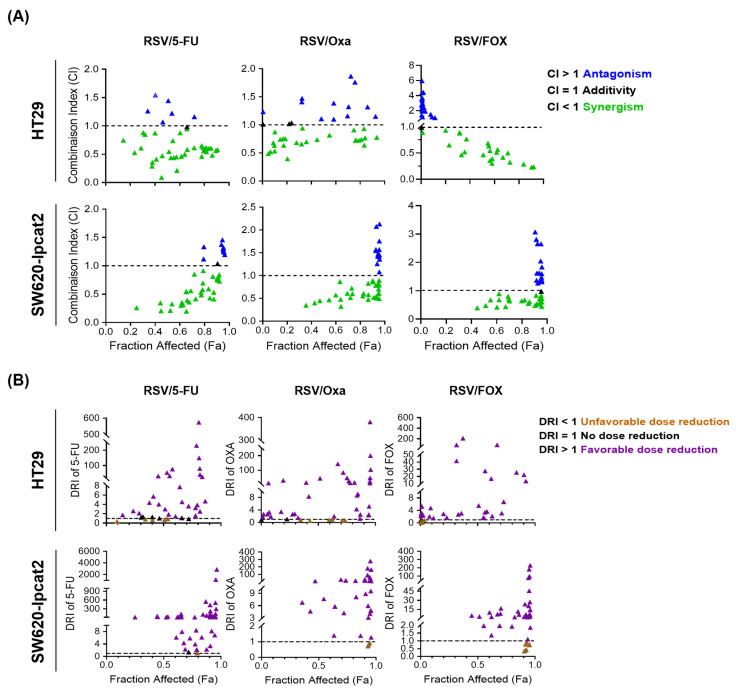
RSV synergizes with conventional chemotherapeutic agents in HT29 cells and SW620-lpcat2 CRC cells. Cells were pretreated with increasing concentrations of resveratrol (RSV) for 24 h before exposure to increasing concentrations of 5-fluorouracil (5-FU), oxaliplatin (OXA), or the FOX (5-FU + OXA, 1:1 ratio) regimen. After 72 h of treatment, cell viability was assessed by crystal violet staining. Absorbances values were normalized to vehicle-treated controls and converted into fraction affected (Fa) values. Drug interactions were analyzed using CompuSyn software according to the Chou–Talalay method. (**A**) Combination index (CI) values for RSV combined with 5-FU, OXA, or FOX in HT29 and SW620-lpcat2 cells as a function of Fa. CI values <1, =1, and >1 indicate synergistic, additive, and antagonistic effects, respectively. (**B**) Dose-reduction index (DRI) values indicating the potential reduction in 5-FU, OXA, and FOX doses when combined with RSV. DRI values >1, <1, and =1 indicate a favorable dose reduction, an unfavorable dose reduction, or no dose reduction, respectively. Results are representative of three independent experiments. The horizontal dashed lines indicate CI = 1 in panel (**A**) and DRI = 1 in panel (**B**).

Because RSV reduced cell viability, induced apoptosis, and altered the expression of key enzymes involved in PC metabolism, we next investigated whether RSV could sensitize chemoresistant CRC cells to conventional chemotherapy. To address this question, HT29 and SW620-lpcat2 cells were pretreated with RSV for 24 h prior to exposure to 5-fluorouracil (5-FU), oxaliplatin (OXA), or the FOX regimen (5-FU + OXA, 1:1 ratio) with fresh medium for an additional 48 h. The effects of this sequential treatment schedule were evaluated using the Chou–Talalay method, which provides a quantitative assessment of drug interactions through the combination index (CI), with CI values <1, =1, and >1 indicating synergistic, additive, and antagonistic effects, respectively [28,29]. The fraction affected (Fa) represents the fraction of cells killed by the treatment, as determined by the crystal violet assay. As shown in Figure 6A, most RSV-based sequential combinations yielded CI values below 1 over a broad range of Fa values in both HT29 and SW620-lpcat2 cells. According to the Chou–Talalay analysis, these values are consistent with synergistic interactions under the sequential treatment schedule used herein. Biologically, however, these findings indicate that RSV pretreatment enhances the cytotoxic activity of 5-FU, OXA and FOX, thereby sensitizing chemoresistant CRC cells to subsequent chemotherapy. To further evaluate the pharmacological relevance of these interactions, the dose-reduction index (DRI) was calculated for each chemotherapeutic agent in combination with RSV. Most combinations yielded DRI values greater than 1 (Figure 6B), indicating that equivalent reductions in cell viability could be achieved using lower concentrations of 5-FU, OXA or FOX following RSV pretreatment.

Collectively, these findings demonstrate that transient RSV pretreatment enhances the subsequent response of chemoresistant CRC cells to conventional chemotherapy and supports its potential use as a chemosensitizing strategy to improve the efficacy of standard treatments.

## 3. Discussion

Our findings show that resveratrol (RSV) decreases cell viability, induces apoptosis, alters the expression of proteins involved in phosphatidylcholine (PC) synthesis and remodeling, modulates lipid droplet (LD) dynamics, and enhances the response to conventional chemotherapeutic agents (5-FU, OXA and FOX) in two resistant CRC models. Combination-index analyses further identified synergistic interactions under the sequential treatment conditions tested. Collectively, these observations support a chemosensitizing effect of RSV and suggest that modulation of PC-metabolism-related proteins may contribute to this response. However, because PC synthesis, remodeling fluxes, and enzyme activities were not directly measured, the present data should not be interpreted as demonstrating direct inhibition of PC biosynthesis. These findings are consistent with previous studies describing RSV as a pleiotropic chemosensitizing compound in CRC, capable of activating cell-death pathways and enhancing the effects of conventional cytotoxic agents in preclinical settings [19]. Beyond its effects on cell survival, our data suggest that RSV acts, at least in part, through changes in proteins associated with PC metabolism. The central role of PC in membrane biogenesis, lipid signaling, and cellular stress adaptation is now well established. PC homeostasis relies on two interconnected pathways: the Kennedy pathway, responsible for de novo PC synthesis, and the Lands cycle, which regulates phospholipid remodeling through lysophospholipid reacylation [5,30]. Within this framework, LPCAT enzymes such as LPCAT1/2 localize to sites involved in LD dynamics and remodeling, whereas CHPT1 catalyzes the terminal step of PC synthesis at the endoplasmic reticulum (ER)/Golgi interface, thereby contributing to the balance between membrane phospholipid supply and neutral-lipid storage [6,27]. In the present study, RSV altered the abundance of proteins associated with both pathways, including CKα, CCTα, CHPT1, and LPCAT2, supporting the hypothesis that RSV may affect several levels of PC metabolic regulation.

The specific role of LPCAT2 in CRC chemoresistance provides an important framework for interpreting our findings. We previously demonstrated that LPCAT2 promotes LD biogenesis and protects CRC cells from chemotherapy-induced ER stress and apoptosis, thereby contributing to resistance to 5-FU and OXA in vitro and in vivo, with parallel changes in immunogenic cell death and CD8^+^ infiltration [8,9]. In SW620-lpcat2 cells, RSV reduced the expression of selected Kennedy pathway enzymes and LPCAT2, while simultaneously promoting a marked and sustained accumulation of LD. Although LD accumulation is commonly associated with tumor aggressiveness and resistance [11], the increase in LD content observed following RSV exposure does not necessarily indicate a protective phenotype. Rather, this response may reflect an adaptive redistribution of cellular lipids toward neutral lipid storage in response to metabolic stress. LD accumulation may also depend on the availability of neutral lipids and phospholipids required to sustain LD biogenesis [11]. The concomitant reduction in PC-metabolism-related proteins could potentially affect phospholipid availability or remodeling; however, no direct causal relationship can be established from the present data. Conversely, HT29 cells displayed a different response to RSV. Despite a transient increase in LD levels at the early time point of 24 h, prolonged RSV exposure did not significantly alter LD content. This observation occurred together with the absence of detectable modulation of LPCAT2 protein expression after 72 h of treatment. This pattern may reflect a distinct adaptive response to RSV-induced metabolic stress in HT29 cells. However, the present experiments do not allow us to determine whether the transient LD increase results from redistribution of lipid fluxes, changes in neutral lipid synthesis, or altered phospholipid availability.

These findings highlight cell-context-dependent responses to RSV rather than demonstrating distinct mechanisms of lipid adaptation. In SW620-lpcat2 cells, 72 h of RSV exposure reduced CKα and CHPT1 protein expression together with LPCAT2 expression, suggesting a concomitant modulation of proteins associated with de novo PC synthesis and PC remodeling. In contrast, HT29 cells displayed more moderate alterations in Kennedy pathway enzymes and no detectable change in LPCAT2 expression. These differences may contribute to the distinct LD profiles observed in the two cellular models, although this hypothesis will require direct functional validation. The Kennedy pathway primarily provides de novo PC required for membrane biogenesis and neutral lipid synthesis, whereas the Lands cycle regulates phospholipid remodeling at LD surfaces and cellular membranes. Compensatory crosstalk between these pathways may therefore contribute to the maintenance of phospholipid homeostasis under RSV exposure, as previously reported in other biological systems [31,32].

**Figure 7:**
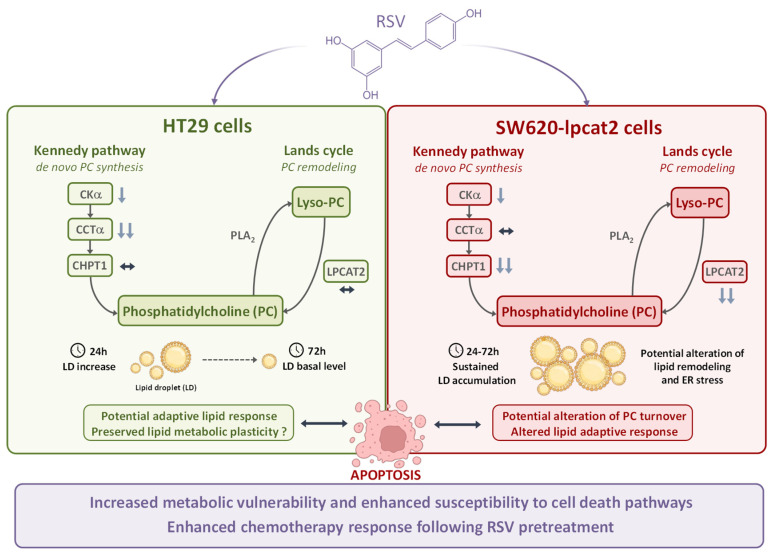
Proposed model illustrating the observed effects of RSV on PC-metabolism-related proteins and lipid droplet dynamics in chemoresistant CRC cells.

Importantly, the present findings resonate with our recent work identifying CHPT1 as a central metabolic driver of chemoresistance in CRC [26]. In that study, CHPT1 overexpression enhanced PC accumulation, altered PC/LPC ratios, and promoted resistance to chemotherapy, whereas pharmacological or functional disruption of the Kennedy pathway improved chemosensitivity. In the present work, RSV markedly reduced CHPT1 expression in SW620-lpcat2 cells, consistent with the possibility that perturbation of PC-metabolism-related processes contributes to RSV-mediated chemosensitization. CHPT1 catalyzes the final and irreversible step of the CDP–choline pathway (Figure 7), thereby contributing to PC availability for membrane biogenesis, lipid signaling, and cellular stress adaptation. LPCAT2, in turn, sustains PC remodeling at cellular membranes and at the LD monolayer, contributing to LD expansion and chemotherapy tolerance. Thus, the concurrent modulation of CHPT1 and LPCAT2 protein expression by RSV may affect complementary aspects of PC homeostasis. However, this interpretation remains hypothetical because neither enzyme activity nor PC metabolic flux was directly measured in the present study.

The concomitant changes in the expression of Kennedy pathway- and LPCAT2-related proteins may have consequences for membrane lipid organization and tumor cell survival. Previous studies have shown that RSV can modify membrane lipid composition and increase intracellular PC levels in CRC cells [33]. In addition, RSV has been reported to interact with phospholipase A_2_ (PLA_2_), thereby limiting phospholipid hydrolysis and altering membrane phospholipid turnover [34]. Such alterations in PC metabolism may affect membrane dynamics and membrane-associated signaling pathways involved in apoptosis and ER stress responses. Consequently, the chemosensitizing effects observed in the present study may not result from a reduction in global LD content, but could be associated with changes in proteins involved in PC homeostasis and membrane phospholipid remodeling. Changes in membrane PC composition could influence membrane fluidity, lipid microdomain organization, and stress signaling pathways, ultimately promoting apoptosis and sensitizing CRC cells to chemotherapy. These potential mechanisms will require validation by targeted lipidomic, enzymatic, and membrane-composition analyses.

Our apoptosis analyses further support the chemosensitizing activity of RSV. RSV induced concentration- and time-dependent increases in apoptotic cell populations together with cleavage of caspase-3 and PARP, consistent with previous reports describing RSV-mediated activation of apoptotic signaling pathways in CRC models [21,22]. Although RSV has also been reported to modulate membrane-associated receptor signaling and death-receptor pathways, our data do not directly address these mechanisms and instead support activation of convergent apoptotic execution pathways [19,21,35]. Our drug-interaction analyses indicate that transient RSV exposure enhances the subsequent response of chemoresistant CRC cells to 5-FU, OXA, and FOX. According to the Chou–Talalay analysis, CI values below 1 observed for several treatment conditions are consistent with schedule-dependent synergistic interactions. From a biological perspective, however, the results are more appropriately interpreted as a priming and chemosensitizing effect of RSV on tumor-cell susceptibility, because RSV was removed before chemotherapy exposure. This interpretation is consistent with the pleiotropic activity of RSV, which has been shown to modulate signaling pathways, redox homeostasis, and metabolic processes involved in cancer-cell survival and therapeutic response [36,37,38]. In the present study, changes in the expression of proteins involved in PC synthesis and remodeling, together with alterations in LD dynamics and apoptosis induction, suggest that modulation of lipid homeostasis may contribute to this chemosensitizing effect. However, the precise mechanisms remain to be established. Several treatment conditions also yielded favorable dose-reduction indices (DRI > 1). These findings are particularly relevant from a translational perspective, as reducing chemotherapy doses while maintaining therapeutic efficacy could potentially limit systemic toxicity and improve treatment tolerability [29]. Similar enhancing effects of RSV on conventional cytotoxic agents, including irinotecan’s metabolite SN38 and oxaliplatin, have previously been reported [35]. The enhanced response to 5-FU- and OXA-based treatments may be associated with the modulation of PC-metabolism-related proteins by RSV. Reduced expression of selected Kennedy pathway enzymes together with altered LPCAT2 expression could potentially influence PC-derived lipid mediators, membrane dynamics, and stress adaptation, while the pro-apoptotic activity of RSV may further favor cell death [3]. Interestingly, chemosensitization was observed in both HT29 and SW620-lpcat2 cells despite distinct LD responses, suggesting that RSV-mediated chemosensitization may occur independently of global LD accumulation, at least in vitro.

Several limitations of the present study should be acknowledged. First, the mechanistic analyses were conducted in HT29 and SW620-lpcat2 cells, which represent distinct genetic backgrounds and resistance contexts. HT29 cells were used as a model of intrinsic resistance, whereas SW620-lpcat2 cells represent an engineered LPCAT2-associated resistant phenotype. Although SW620-Ctl cells provided an isogenic control in the viability experiments, they were not included in all downstream mechanistic analyses. Consequently, differences observed between HT29 and SW620-lpcat2 cells cannot be attributed exclusively to LPCAT2 expression, and the specific contribution of LPCAT2 to the effects of RSV will require validation in additional isogenic models. Second, the present study assessed the abundance of proteins involved in the Kennedy pathway and the Lands cycle but did not directly measure their catalytic activities, PC and LPC abundance, or metabolic flux through these pathways. Changes in protein expression should therefore not be interpreted as direct evidence of pathway inhibition or altered PC synthesis and remodeling. Dedicated substrate-based enzymatic assays combined with targeted LC-MS/MS, comprehensive lipidomics, and isotope-tracing approaches will be required to confirm the functional consequences of RSV treatment on PC metabolism. Third, LD accumulation was assessed globally, whereas potential changes in LD composition, including triglyceride and cholesterol ester content, were not investigated. Previous studies suggest that alterations in LD lipid composition may critically influence the balance between survival and apoptosis in CRC cells [39].

These considerations are particularly important because the effects observed in vitro are likely to depend on RSV exposure and intracellular bioavailability. Although RSV exhibits limited systemic bioavailability due to rapid conjugation [40,41], previous studies have shown that RSV and its metabolites accumulate in colorectal tissues at biologically active concentrations [42] where intracellular deconjugation mechanisms may locally regenerate free RSV [43]. Nevertheless, in vivo validation will be essential to determine whether the chemosensitizing effects observed in vitro can be reproduced under physiological conditions, in which RSV pharmacokinetics, the predominance of conjugated metabolites in the colorectum, and delivery strategies are likely to influence therapeutic outcomes [35]. In this context, nanoparticle- or liposome-based delivery systems may represent promising strategies to improve RSV stability, prolong circulation, and optimize tumor targeting [44]. More importantly, co-encapsulation of RSV with conventional chemotherapeutic agents could synchronize pharmacokinetics and further enhance chemosensitization while limiting off-target toxicity [45,46]. In CRC, where LPCAT2-driven phospholipid remodeling contributes to metabolic adaptation and chemoresistance, such delivery systems may reinforce RSV-mediated metabolic stress and improve therapeutic efficacy.

## 4. Materials and Methods

### 4.1. Cell Culture

Human colorectal cancer cell lines HT29 and SW620 were obtained from the American Type Culture Collection (ATCC), Manassas, VA, USA. SW620 cells stably overexpressing LPCAT2 (SW620-lpcat2) and corresponding empty vector control cells (SW620-Ctl) were generated as previously described using the pCMV6-AC-GFP retroviral vector containing the human LPCAT2 gene (h-LPCAT2; NM_017839) or the empty vector, respectively [8]. Cells were cultured in Dulbecco’s Modified Eagle Medium (DMEM) supplemented with 10% fetal bovine serum (FBS, Dutscher, Brumath, France) and 1% penicillin–streptomycin (P06-07300, Dutscher, Brumath, France) and maintained at 37 °C in a humidified incubator with 5% CO_2_. Mycoplasma potential contamination was routinely assessed by using the MycoAlert kit (Lonza, Levallois-Perret, France).

### 4.2. Drug Treatments

Resveratrol (RSV, #R5010, Sigma Aldrich, St. Quentin Fallavier, France) stock solution was prepared at 100 mM in absolute ethanol and stored at −20 °C. Oxaliplatin (OXA) and 5-fluorouracil (5-FU) (Accord Healthcare Limited, Lille, France) were provided as clinical-grade infusion solutions and diluted in culture medium to the indicated concentrations. In all experimental conditions, the final solvent concentration did not exceed 0.1% (*v*/*v*). Vehicle-treated cells were used as controls.

### 4.3. Cell Viability Assays

HT29, SW620-Ctl, and SW620-lpcat2 cells were seeded in 96-well plates and allowed to adhere for 24 h. Cells were then treated with increasing concentrations of RSV (0–500 µM) for 24, 48, or 72 h. Cell viability was assessed using crystal violet staining (#HT90132, Sigma-Aldrich, St. Quentin Fallavier, France), which was selected because it quantifies the remaining adherent cellular biomass independently of cellular metabolic activity, thereby minimizing potential bias associated with metabolic effects with resveratrol. After washing and drying, bound dye was solubilized in 33% acetic acid, and absorbance was measured at 590 nm using an EnVision^®^ Multimode Plate Reader (PerkinElmer, Villebon-sur-Yvette, France). Viability was normalized to vehicle-treated controls. Half-maximal inhibitory concentrations (IC_50_) values were calculated by four-parameter nonlinear regression using GraphPad Prism v10.6.0 (GraphPad Software, Boston, MA, USA).

### 4.4. Western Blot Analysis

Western blot analyses were performed on protein extracts from HT29 and SW620-lpcat2 cells treated under the indicated conditions. Cells were washed twice with PBS and lysed on ice in RIPA buffer (50 mM Tris-HCl, 150 mM NaCl, 0.1% SDS, 0.5% sodium deoxycholate, 1% NP-40, pH 8.0) supplemented with protease inhibitor cocktail (#11697498001, Roche, Boulogne-Billancourt, France), 50 mM sodium fluoride, and 100 µM phenylmethylsulfonyl fluoride (PMSF). Lysates were clarified by centrifugation (20 min, 16,000× *g*, 4 °C), and protein concentrations were determined using the QuantiPro™ BCA Assay Kit (#QPBCA, Sigma-Aldrich, St. Quentin Fallavier, France).

Equal amounts of protein (30–50 µg) were mixed with Laemmli sample buffer and heated at 95 °C for 5 min before separation by SDS-PAGE using 7.5%, 10%, or 12.5% TGX Stain-Free™ FastCast™ gels (#1610185, #1610183, #1610185, Bio-Rad, Marnes-la-Coquette, France). Proteins were transferred to nitrocellulose membranes (#1704271, Bio-Rad) using the Trans-Blot^®^ Turbo™ system (#1704150, Bio-Rad). Membranes were blocked for 1 h in either 5% bovine serum albumin (BSA) or 5% skimmed milk diluted in PBS containing 0.1% Tween-20 (PBS-T), followed by overnight incubation at 4 °C with primary antibodies purchased from Cell Signaling Technology (Ozyme, Saint-Cyr-l’École, France), Abcam (Cambridge, UK), Sigma-Aldrich (St. Quentin Fallavier, France) or Santa Cruz Biotechnology (Heidelberg, Germany), as detailed in Table 2. HRP-conjugated secondary antibodies (#115-035-146 and #111-035-144, Jackson ImmunoResearch, West Grove, PA, USA) were then incubated for 1 h at room temperature.

Protein detection was performed using enhanced chemiluminescence reagents (Bio-Rad) and a ChemiDoc™ MP imaging system (Bio-Rad). Densitometric analyses were carried out using Image Lab™ software v6.0.1 (Bio-Rad). HSC-70 was used as loading control, and protein expression levels were normalized to vehicle-treated controls.

### 4.5. Flow Cytometry Analysis

Flow cytometry analyses were performed using an LSRFortessa™ cytometer and FACSDiva software v8.0 (BD Biosciences, Le Pont-de-Claix, France). These experiments were performed at the Imaflow facility part of the US58 BioSanD and analysis was done on instruments in the Imaflow part of the US58 BioSanD. Data were analyzed with FlowJo software v10.0.7 (Tree Star, Ashland, OR, USA).

#### 4.5.1. AnnexinV/7-AAD Staining

Apoptosis was evaluated using Annexin V-FITC (#556419, BD Biosciences) and 7-aminoactinomycin D (7-AAD, #559925, BD Biosciences) staining according to the manufacturer’s instructions. HT29 and SW620-lpcat2 cells were seeded in 12-well plates and treated as indicated for 24, 48, or 72 h. Both floating and adherent cells were collected, washed twice with cold PBS 1×, and resuspended in binding buffer (#556454, BD Biosciences) containing Annexin V-FITC and 7-AAD. After incubation for 15 min at room temperature in the dark, 200 µL of binding buffer was added, and samples were analyzed by flow cytometry. Unstained cells and single-stained controls were used for compensation settings.

#### 4.5.2. BODIPY 493/503 Staining

HT29 and SW620-lpcat2 cells were seeded in 12-well plates and treated under the same conditions as described for Annexin V/7-AAD staining. After 24, 48, or 72 h of treatment, floating and adherent cells were collected, washed with PBS 1×, and stained with BODIPY™ 493/503 (D3922, Molecular Probes, Eugene, OR, USA) diluted 1:2000 in PBS 1× for neutral lipid detection together with Fixable Viability Stain 700 (FVS700; #564997, BD Biosciences) diluted 1:5000 to exclude dead cells. After 20 min incubation at room temperature in the dark, cells were washed twice with PBS 1× and resuspended prior to analysis. Lipid droplet content was quantified by measuring the median fluorescence intensity (MFI) of BODIPY 493/503 in viable FVS700-negative cells.

### 4.6. Immunofluorescence Staining

HT29 and SW620-lpcat2 cells were seeded on coverslips in 24-well plates and treated as described above. After treatment, cells were washed three times with TBS 1× and fixed with 4% paraformaldehyde (PFA; #1004968350, Sigma-Aldrich) for 10 min at room temperature. Following two washes with TBS 1×, lipid droplets were stained with BODIPY™ 493/503 (D3922, Molecular Probes; 1:2000) for 45 min at room temperature. Coverslips were subsequently washed with TBS 1× followed by ultrapure water before mounting using ProLong™ Gold Antifade reagent containing DAPI (#P36941, Thermo Fisher Scientific, Waltham, MA, USA). Images were acquired with an AxioImager M2 microscope (ZEISS, Rueil Malmaison, France) and analyzed using Icy software (v3, Institut Pasteur and France-BioImaging).

### 4.7. Combination Index Analysis

HT29 and SW620-lpcat2 cells were seeded in 96-well plates and incubated overnight. Cells were pretreated for 24 h with increasing concentrations of RSV, followed by exposure to increasing concentrations of 5-fluorouracil (5-FU), oxaliplatin (OXA), or the FOX regimen in fresh medium for an additional 48 h. Cell viability was assessed using crystal violet staining as described in Section 4.3. Drug interactions were analyzed according to the Chou–Talalay method [28] as previously described [47]. Combination index (CI) and dose-reduction index (DRI) values were calculated using CompuSyn software (version 1.0; ComboSyn Inc., Paramus, NJ, USA) over a range of fraction affected (Fa) values from 0.05 to 0.90. CI values <1, =1, and >1 indicate synergistic, additive, and antagonistic effects, respectively. DRI values > 1 indicate favorable dose reduction. CI–Fa and DRI–Fa plots were generated using CompuSyn software.

### 4.8. Statistical Analysis

Data are presented as mean ± standard error of the mean (SEM), from at least three independent experiments with a minimum of three replicates per condition. Statistical analyses were performed using GraphPad Prism v10.6.0 (GraphPad Software, Boston, MA, USA).

Continuous variables were analyzed using one-way ANOVA, or two-way ANOVA followed by Tukey’s multiple comparison test, as indicated in the figure legends. All statistical tests were two-tailed, and *p* values < 0.05 were considered statistically significant (ns, not significant; * *p* <0.05, ** *p* <0.01, *** *p* <0.001, **** *p* <0.0001).

## 5. Conclusions

Collectively, our findings support the involvement of phosphatidylcholine metabolic pathways in the chemosensitizing effects of RSV in CRC cells and suggest that simultaneous disruption of de novo PC biosynthesis and PC remodeling pathways may represent an effective strategy to overcome chemoresistance. From a translational perspective, RSV appears capable of reproducing some effects associated with disruption of CHPT1- and LPCAT2-related pathways previously implicated in CRC resistance mechanisms. In this context, modulation of Kennedy pathway enzymes together with alterations in LPCAT2 expression may contribute to RSV-induced metabolic stress, apoptosis, and chemosensitization in resistant CRC models. Altogether, these findings highlight PC metabolism as a therapeutically actionable vulnerability in CRC and support further investigation of RSV-based metabolic adjuvant strategies for chemoresistant CRC.

## Figures and Tables

**Table 1:** Half-maximal inhibitory concentrations (IC_50_) of resveratrol (RSV) in colorectal cancer (CRC) cell lines. Data are presented as mean ± SEM from three independent experiments. *p* values were determined using a one-way ANOVA followed by Tukey’s multiple comparison test. * *p* < 0.05: SW620-Ctl vs. SW620-lpcat2; ^$$$^ *p* < 0.001: HT29 vs. SW620-Ctl.

Time	Inhibitory Concentration 50% (IC_50_) (μM of RSV)		
	HT29	SW620-Ctl	SW620-lpcat2
24 h	157.4 ± 5.38	108.9 ± 7.44 *	169.5 ± 20.31
48 h	40.05 ± 3.61 ^$$$^	13.01 ± 1.57 *	30.04 ± 5.04
72 h	22.39 ± 2.63	8.75 ± 1.77	28.39 ± 12.10

**Table 2:** Antibodies.

Antibody	Provider	Reference	Isotype	Dilution
PARP	Cell Signaling Technology	#9542	Rabbit	1:1000
Caspase 3	Cell Signaling Technology	#14220	Rabbit	1:1000
CHKA	Sigma-Aldrich	HPA024153	Rabbit	1:1000
LPCAT2	Sigma-Aldrich	SAB4502278	Rabbit	1:1000
CCTα. clone D18B6	Cell Signaling Technology	#6931	Rabbit	1:1000
CHPT1	Abcam	ab107425	Rabbit	1:1000
HSC-70	Santa Cruz Biotechnology	sc-7298	Mouse	1:1000

## Data Availability

Data are available upon reasonable request.
